# Minute-Scale Synthesis of Nano Silicalite-1 Zeolites

**DOI:** 10.3389/fchem.2022.860795

**Published:** 2022-04-08

**Authors:** Changsheng Zhang, Shaoqi Chu, Jie Jiang, Jinchong Zhao, Song Wen, Bing Sun, Wei Xu

**Affiliations:** ^1^ SINOPEC Research Institute of Safety Engineering Co., Ltd., Qingdao, China; ^2^ College of Chemical Engineering, Qingdao University of Science and Technology, Qingdao, China

**Keywords:** zeolite, silicalite-1, fast synthesis, green chemistry, O_2_ removal

## Abstract

Synthesis of zeolites in more efficient and greener methods is of great significance in both industrial and academic fields. However, the relative long time for zeolite crystallization and much consumption of water solvent make the target challengeable. Herein, a route for ultrafast synthesis of nano Silicalite-1 zeolites in 10 min with much less water consumption has been developed. Comprehensive characterizations, i.e., X-ray powder diffraction, N_2_ sorption, scanning electron microscope, and NMR, confirm the high quality of such obtained Silicalite-1 zeolites. In the catalytic deoxygenation of O_2_-containing ethylene (mixture of O_2_ and ethylene), these reported Silicalite-1 zeolite samples show the comparable performance with the conventional Silicalite-1 zeolites synthesized under hydrothermal conditions. This research therefore provides a new trial toward the ultrafast synthesis of zeolite materials in an environment-friendly route.

## Introduction

Zeolites materials with ordered pore structures have been widely used in catalysis and adsorption because of the rich pore structures and outstanding stability (Beck et al., 1992; Davis., 2002; Majano et al., 2009; Meng and Xiao, 2014; Ke et al., 2018; Chen et al., 2020; Campo et al., 2021; Zhai et al., 2021; Peng et al., 2022). Among all the emerged topologies, zeolites with MFI topology have always been drawing people’s attention due to their unique structure, composed of interconnected 10-member-ring straight and sinusoidal channels (Haw et al., 2003; Bjorgen et al., 2007; Olsbye et al., 2012; Li et al., 2014; Tian et al., 2015; Zhang et al., 2018a; Dai et al., 2018; Zhang et al., 2018b; Ma et al., 2019; Chen et al., 2022; Zhou et al., 2022; Zhang et al., 2018c). Typically, aluminosilicate MFI zeolites show quite excellent performance in petrochemical and coal chemical industrial. Titanosilicate MFI zeolites have been widely used in fine chemical engineering, such as the epoxidation of propene. It is proven that the pure silica MFI zeolite, Silicalite-1, could be successfully applied in catalysis and gas separation. However, it usually takes several hours or even several days to obtain these zeolite materials with high crystallinity, consuming much energy and increasing the comprehensive costs. In addition, the traditional hydrothermal synthesis of zeolite consumes large amounts of water solvents and has low space yields for zeolite products. How to make the industrial production of zeolites more efficient and greener is of great significance in both the industrial and academic fields.

To shorten the time consumption for zeolite synthesis, scientists developed various methods in the past several decades. Okubo, Liu, and co-workers proposed the continuous flow synthesis of zeolites, which made significant contribution to the zeolite fast synthesis (Liu et al., 2016). The researchers also developed several methods to accelerate the crystallization of zeolites, such as APO-5 (Liu et al., 2014) and Silicalite-1 (Liu et al., 2016), where the Silicalite-1 zeolite could be synthesized within 10 min in hydrothermal atmosphere with the assistance of 2-day aging period at room temperature. Yu et al. reported the ultrafast synthesis of SAPO-34 samples within 10 min (Sun et al., 2015). At the same time, microwave-assisted synthesis (Rao et al., 1999; Lin et al., 2010; Mohanned et al., 2015), dry gels conversion synthesis (Rao and Matsukata, 1996; Cai et al., 2010; Liu et al., 2021), and high temperature synthesis (Bian et al., 2017; Bian et al., 2018) have been proposed to obtain the zeolite products with higher efficiency. However, most of the reported methodologies are occupied in hydrothermal conditions or with relatively high energy cost. Fast synthesis of zeolite with high integrated efficiency (herein, less water consumption and less energy cost) still remains a challenge.

Recently, Xiao and co-workers proposed the solvent-free synthesis of zeolites, followed by the successful synthesis of silicoaluminophosphate and pure silica zeolites (Ren et al., 2012; Jin et al., 2013; Wu et al., 2014; Wu et al., 2019). These reports opened up a new research field in zeolite synthesis, exhibiting us a promising method to make zeolite production more environment-friendly. However, there is still no success for synthesis of zeolite with high crystallinity *via* this methodology within 10-min time scale yet.

Herein, we report a successful solvent-free synthesis of nano Silicalite-1 zeolites in 10 min with no additional water solvents. To our best knowledge, this is the fastest speed for Silicalite-1 synthesis achieved in the solvent-free atmosphere. What is more, there is no aging period needed for nucleation process, further decreasing the time consumption for zeolite synthesis. The proper H_2_O/SiO_2_ ratio and precisely adjusted alkalinity play the critical role for the fast obtaining of these zeolite samples. The samples are designed as F-S-1. In the deoxygenation of O_2_-containing organic gases (mixture of O_2_ and organic gases), which is an important reaction in intrinsically safe disposal of O_2_-containing organic gases to decrease explosion risk, Pd-supported F-S-1 catalyst (Pd/F-S-1) exhibits the comparable catalytic performance with the conventional Silicalite-1 samples synthesized in hydrothermal conditions.

## Materials and Methods

### Materials

Silica gel was purchased from Aladdin. NaOH, tetrapropylammonium hydroxide (TPAOH of 40 wt.%), tetraethyl orthosilicate (TEOS), NH_4_Cl, and PdCl_2_ were received from Sinopharm Chemical Reagent Co. Commercial Silicalite-1 zeolite samples are supplied by Sinopec. Deionized water was made in the laboratory. All the materials were used as-received without any further treatment.

### Synthesis Process

*Ultrafast synthesis of F-S-1.* F-S-1 zeolites are synthesized in a stainless autoclave at the temperature of 200°C with the molar ratio of 100SiO_2_/17.7TPA^+^/3.7Na_2_O/7S-1 seed. In a typical run, 5.0 g of silica gel, 0.25 g of NaOH, and 0.35 g of Silicalite-1 seeds were mixed in a mortar, followed by a carefully grinding process at room temperature. Then, 7.5 g of TPAOH (40 wt.%) was added and grinded with the mixtures. The finally obtained mixtures are transferred into a sealed autoclave and heated at 200°C for 10 min. After centrifugation at room temperature and dry at 80°C, the obtained zeolite samples are calcined at 550°C for 5 h to get the samples with open channels. Sodium ions are removed by ion exchange, followed by calcined at 550°C for 3 h. The amount of water and NaOH in the starting gels could be adjusted when investigating their influences over crystallization. To decrease the influence of heat transfer during synthesis, all the samples are crystallized in a stainless autoclave (shown by Supplementary Figure S1) and heated by oil bath.

### Synthesis of Conventional Silicalite-1 Zeolites

Conventional Silicalite-1 samples are synthesized according to the reported literature with partial modifications with the mol ratio of 100SiO_2_/132TPA^+^/3068H_2_O (Zhang et al., 2018). In a typical run, 5.0 g of TPAOH (20 wt.%) is added into 5.0 g of distilled water. After fully dissolved, 3.2 g of TEOS was added. After stirring for 24 h, the gel was transferred into an autoclave and heated at 180°C for 24–48 h. The organic templates were removed *via* calcination at 550°C for 5 h. The obtained zeolite samples are designed as C-S-1. Such obtained Silicalite-1 zeolites are used as seeds for the F-S-1 zeolite synthesis.

### Synthesis of Pd-Supported Catalyst

The Pd-supported catalyst is obtained by equivalent-volume impregnation, where Pd-supported F-S-1 is designated as Pd/F-S-1 and the Pd-supported C-S-1 is designated as Pd/C-S-1. The amount of the Pd species is 0.5 wt.%.

### Characterization

X-ray powder diffraction (XRD) patterns were measured with a Rigaku Smartlab X-ray diffractometer (45 kV, 200 mA), step size at 0.02° and counting time at using CuK∝ radiation. The morphology of these samples was observed on Hitachi S4800 scanning electron microscope (SEM). The samples were ultrasonic treated for 30 min before SEM characterization. High-resolution transmission electron microscopy (HR-TEM) images of the samples were obtained on a JEM-2100F transmission electron microscope. The calcined samples were dispersed in absolute ethyl alcohol and ultrasonic treated for 20 min before TEM characterization. ^29^Si magic-angle spinning (MAS) NMR spectrum was recorded on a Bruker AVANCEIII 400M spectrometer. The nitrogen sorption isotherms at the temperature of liquid nitrogen were measured using a Micromeritics ASAP 2460. The ionized and calcined zeolite samples were degassed in 200°C for 12 h before measured at temperature of liquid nitrogen.

### Catalytic tests

The catalytic deoxygenation of O_2_-containing ethylene (mixture of O_2_ and ethylene) was performed with a fixed-bed tubular steel reactor. After 1.0 g of catalyst (20–40 mesh) was loaded in the middle of tubular steel between two layers of quartz wool, it was pretreated in flowing H_2_ at 200°C for 2 h and cooled down to a reaction temperature of 80°C. The reaction was carried out at 0.3 MPa with the feed gases containing 97% of ethylene and 3% of O_2_. Conversion of O_2_ was analyzed on-line using an Agilent 7890B gas chromatograph equipped with a TCD detector and a PLOT-Q capillary column.

## Results and Discussions

**FIGURE 1:**
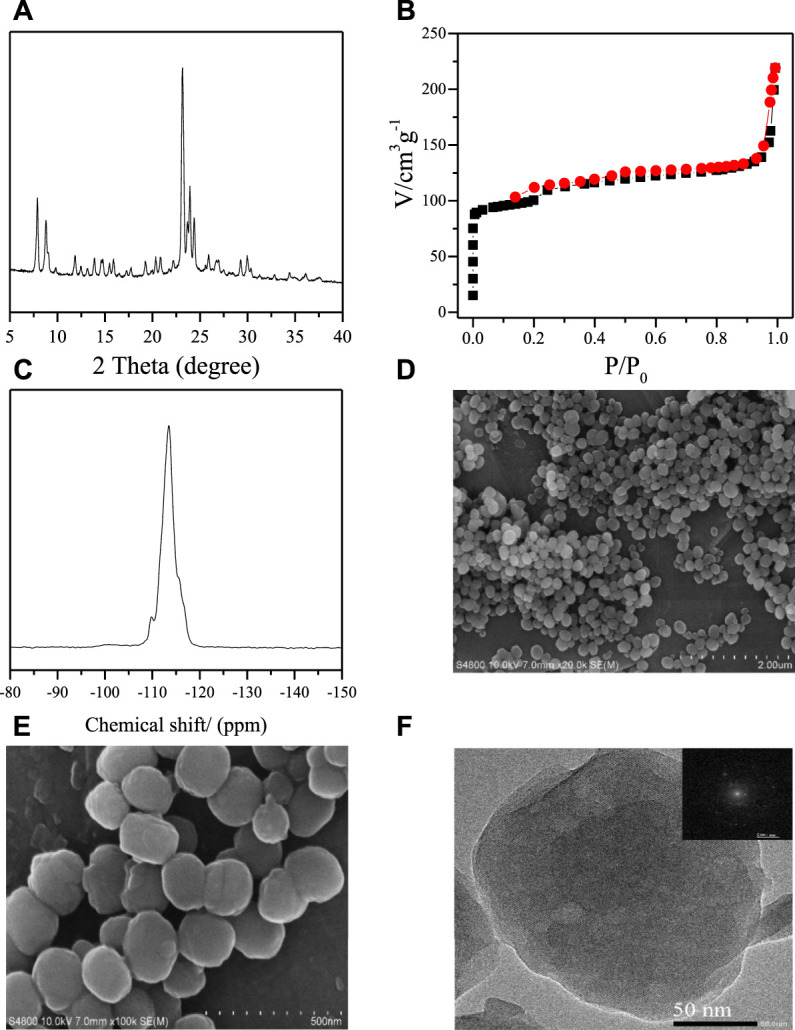
**(A)** XRD pattern, **(B)** N_2_-sorption isotherms, **(C)** ^29^Si MAS NMR spectrum **(D,E)** SEM images, and **(F)** high-magnification TEM image of obtained F-S-1 (inserted in the TEM image is the SAED pattern taken from an entire particle).

To decrease the influence of heat transfer, all the samples are crystallized in a stainless autoclave (Supplementary Figure S1) and heated by oil bath in this research. Figure 1 shows the XRD pattern, N_2_ sorption curves, ^29^Si MAS NMR spectra, SEM images, and TEM image with high resolution of thus obtained S-1 zeolite samples. The XRD pattern shows the typical peaks associated to the MFI topology in the range of 5^**°**^–40^**°**^ (Baerlocher et al., 2007). The N_2_-sorption curves give a sharp increase at relative pressure less than 0.01, which is due to the fast N_2_ filling in the micropores (Sel et al., 2006). The zeolite sample gives the corresponding BET surface and micropore volume at 392 m^2^/g and 0.105 cm^3^/g, respectively. It is interesting to find a gradually increase in the range of 0.2 < P/P_0_ < 0.9, which indicates that there may be mesopores inside the zeolite crystals. The steep increase at P/P_0_ > 0.9 should be assigned to pores caused by the aggregation of the zeolite crystals. Figure 1C shows the ^29^Si NMR spectrum of the obtained the S-1 zeolites. We could find a strong band centered at −113 ppm representing the 4-coordinative Si species [Si(SiO)_4_] and a weak band at −103 ppm assigned to the Q_3_ silica species [(SiO)_3_(OH)]. Figures 1D,E show the SEM images with different magnifications. We could clearly see the aggregation phenomenon of the zeolite crystals in the lower-magnification image (Figure 1D), which is consistent with the results of N_2_ sorption curves. From the SEM image with higher magnification (Figure 1E), we could see that the well-crystallized zeolite crystals show quite uniform crystal size at about 200 nm with the sphere morphology. The high-resolution TEM image (Figure 1F) confirms the high crystallinity of these obtained S-1 zeolite crystals, combined with the XRD, N_2_ sorption, ^29^Si MAS NMR spectrum, and SEM results. In addition, it is interesting to find the mesopores inside the single zeolite crystal, which shows the corresponding information with the N_2_ sorption. The inserted SEAD figure is taken from an entire zeolite crystal, exhibiting the single crystal nature of the obtained zeolite samples.

**FIGURE 2:**
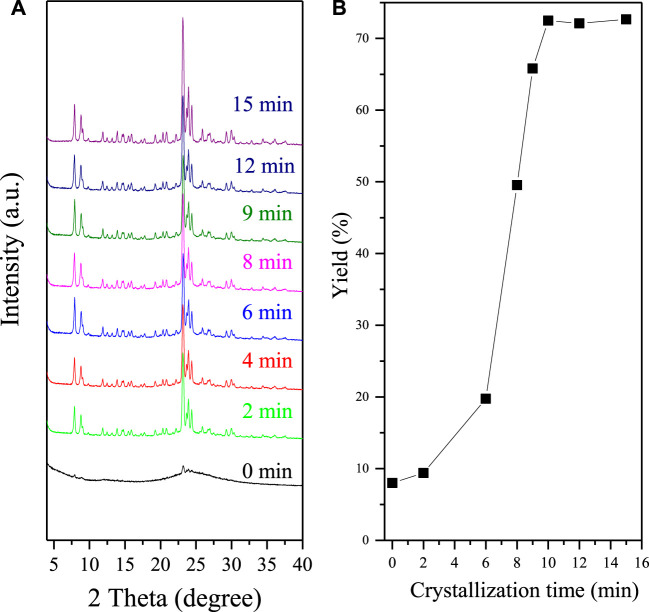
**(A)** XRD patterns of F-S-1 zeolite with crystallization time at (a) 0, (b) 2, (c) 6, (d) 8, (e) 9, (f) 10, (g) 12, and (h) 15 min. **(B)** Dependence of product yields over crystallization time.

To better understand the synthesis nature, the crystallization process of F-S-1 is systematically investigated by XRD and SEM, shown by Figure 2 and Supplementary Figure S2. Figure 2A shows the XRD patterns of the Silicalite-1 zeolite with different crystallization time, whereas Figure 2B shows the zeolite product yields with the corresponding crystallization time. All these samples are obtained after crystallization and centrifugation, except that the sample with crystallization time at 0 min is directly dried after grinding and mixing of the raw materials. Without crystallization, Figure 2Aa shows the weak diffraction peaks related to the MFI topology, which is attribute to the seed crystals (the XRD pattern and SEM images of the S-1 seeds are shown by Supplementary Figures S3, S4). Interestingly, we find that the samples after crystallization for 2 min show quite strong diffraction peaks associated with MFI topology. However, the yield of these samples is quite low at only 9%. At the same time, we could only see a little product sticked on the surface of zeolite seeds from the SEM images shown by Supplementary Figure S2B. This phenomenon means that most of the raw materials are dissolved in the liquid phase during centrifugation process. The photos of the samples after crystallization and centrifugation are shown by Supplementary Figure S5. As crystallization time increase, the product yields gradually increased. For instance, product yields increase to 72% after crystallization of 10 min. Correspondingly, we could see more new zeolite crystals appear in the SEM images (Supplementary Figure S2). Meanwhile, the yield of F-S-1 samples keeps constant as crystallization time goes by after 10 min, which in our opinion represents that the crystallization process has been finished.

It is interesting to obtain these well-crystallized Silicalite-1 samples within so short time. Supplementary Figure S5 shows the photos of the obtained samples after centrifugation with different crystallization time. We could find that most of the solid materials (except the S-1 seeds) are transferred into soluble phase within 2 min, which is an important activation process for zeolite synthesis. The super high alkalinity (compared with the hydrothermal method) should make the positive influence over the fast activation of solid raw materials. At the same time, the influence of water and alkalinity over crystallization is carefully investigated. Interestingly, it takes longer time for Silicalite-1 crystallization, with higher H_2_O/SiO_2_ ratio, such as 15 (Supplementary Figures S6, S7). This phenomenon is similar to our previous research that MFI zeolite could be synthesized within shorter time with lower H_2_O/SiO_2_ ratio, where the system shows faster nucleation rate (Zhang et al., 2018). Supplementary Figure S8 shows the influence of alkalinity over zeolite crystallization, which indicates too high alkalinity is not favorable for zeolite crystallization. Hence, proper alkalinity, precise adjustment of H_2_O/SiO_2_ ratio, and the fast heat transfer make the comprehensive contributions to the ultrafast synthesis of the F-S-1 zeolite.

**FIGURE 3:**
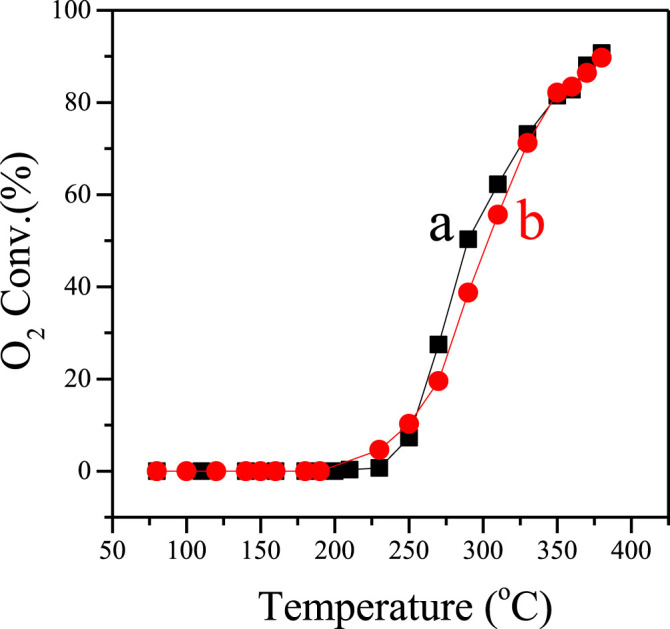
Dependence of O_2_ conversion on temperature over the (a) Pd/F-S-1 and (b) Pd/C-S-1 catalysts.

Figure 3 shows the performance of Pd (0.5 wt.%)–supported F-S-1 and C-S-1 catalysts in the catalytic deoxygenation of oxygen-containing organic gases (mixture of ethylene and O_2_ as representatives herein), which is an important process for intrinsic-secure treatment of oxygen-containing organic gases in chemical engineering. Apparently, Pd/F-S-1 and Pd/C-S-1 show quite similar O_2_ conversion at the same temperature, where the two catalysts could both achieve about 90% O_2_ conversion at 380°C. Considering the super high time-space yield and less water consumption, the F-S-1 zeolite samples show quite promising potentiality in practical industrial application compared to the conventional C-S-1 samples obtained from the hydrothermal method.

In summary, Silicalite-1 zeolites with high crystallinity have been successfully synthesized within 10 min without additional water solvents. In the catalytic deoxygenation of oxygen-containing ethylene, Pd-supported F-S-1 gives the comparable catalytic performance with the C-S-1 synthesized in the hydrothermal conditions. Compared with the conventional hydrothermal zeolite synthesis methods reported in the literatures, the route developed in this research shows obvious advantages in the following aspects: 1) much less consumption of water resource due to the no additional water solvents; 2) much higher space-time-yields. The raw materials could occupy most of the space in the autoclaves, making contribution to the less occupation of land for zeolite production. More importantly, the ultrafast synthesis within 10 min greatly saves the time consumption for zeolite synthesis; 3) relatively low consumption of organic templates. In this search, F-S-1 zeolites with high crystallinity are successfully obtained with TPA^+^/SiO_2_ at 0.17, much lower than that of C-S-1, which agrees with our previous report that high nutrients concentration in the starting gels have positive effect over the nucleation rate for MFI zeolite synthesis (Zhang et al., 2018). The combination of these advantages makes it possible for the potential industrial application of these Silicalite-1 zeolites in the future.

## Data Availability

The original contributions presented in the study are included in the article/Supplementary Material, further inquiries can be directed to the corresponding authors.
