# Supplementary material for: Minute-Scale Synthesis of Nano Silicalite-1 Zeolites
Source: Front Chem. 2022 Apr 8;10:860795. doi: 10.3389/fchem.2022.860795 (PMC9024212; doi:10.3389/fchem.2022.860795)
Supplement: Supplementary file 1 [file DataSheet1.pdf]

Supplementary Material

Minute-scale Synthesis of Nano Silicalite-1 Zeolites

Changsheng Zhang [1,*], Shaoqi Chu [1,2], Jie Jiang [1,*], Jinchong Zhao [1], Song Wen [1], Bing Sun [1], Wei Xu [1,*]

[1]SINOPEC Research Institute of Safety Engineering Co., Ltd. Qingdao, 266100, China

[2]College of Chemical Engineering, Qingdao University of Science and Technology, Qingdao, 266042, China

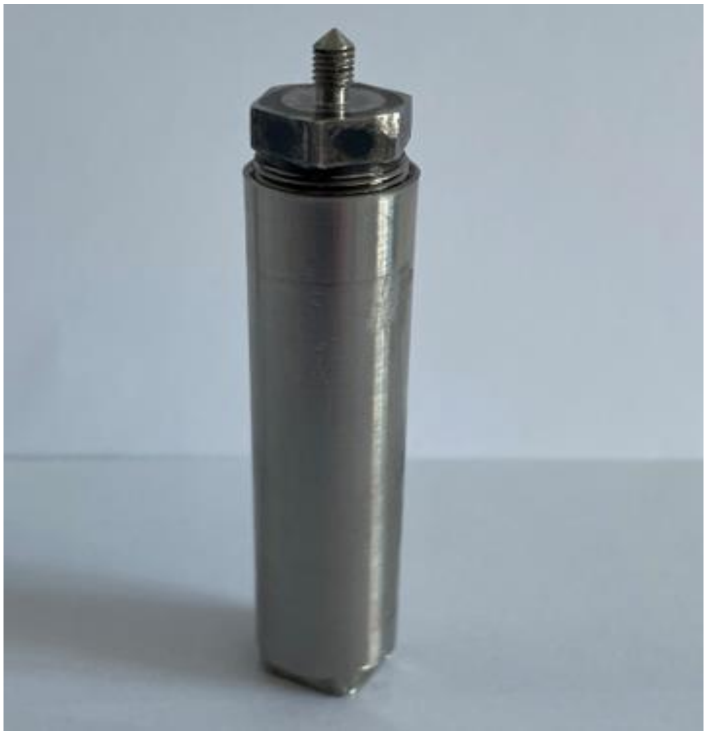

Figure S1. Photo of the stainless autoclave.

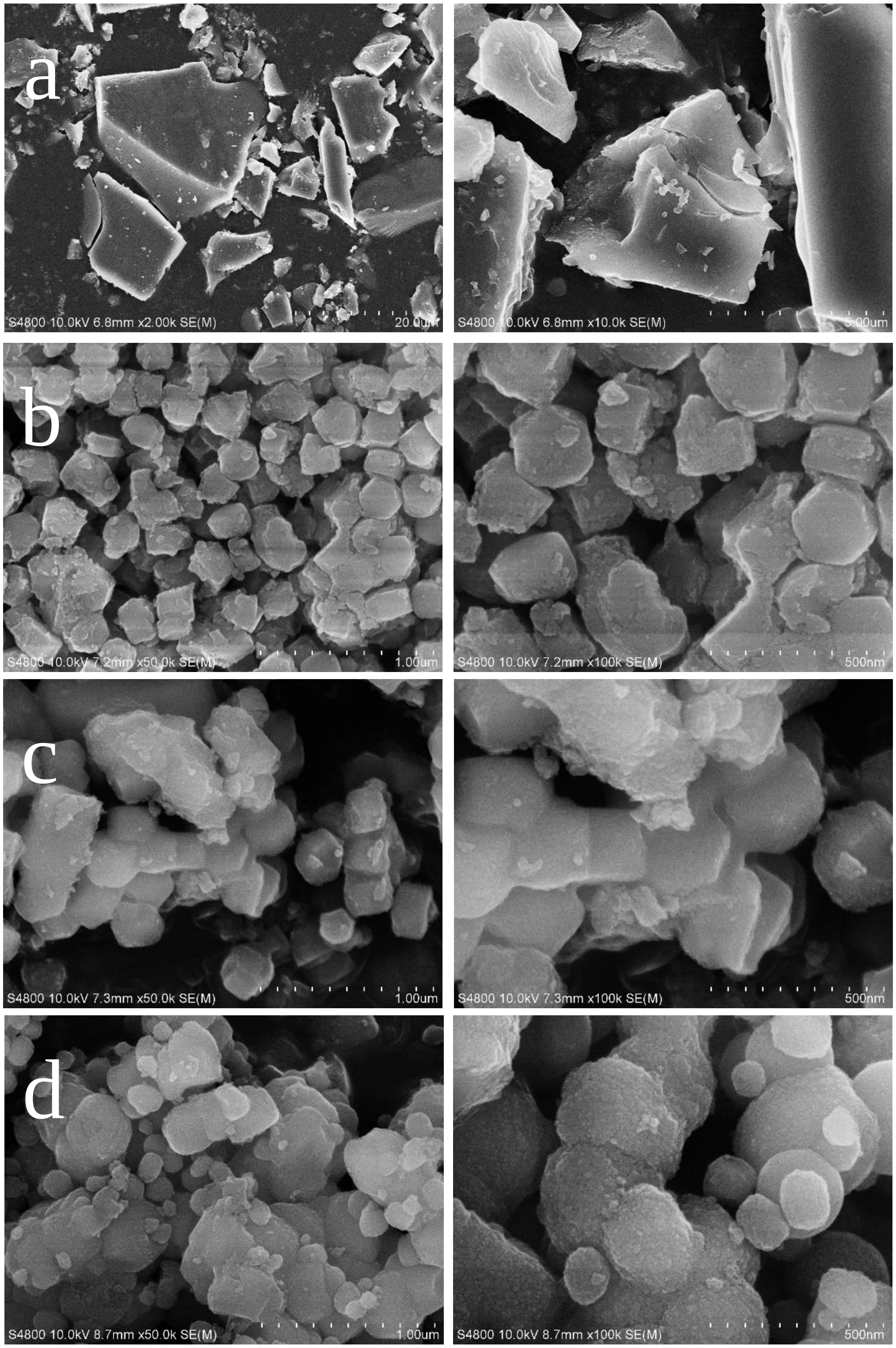

Figure S2. SEM images of S-1 with crystallization time at (a) 0, (b) 2, (c) 6, and (d) 8 min.

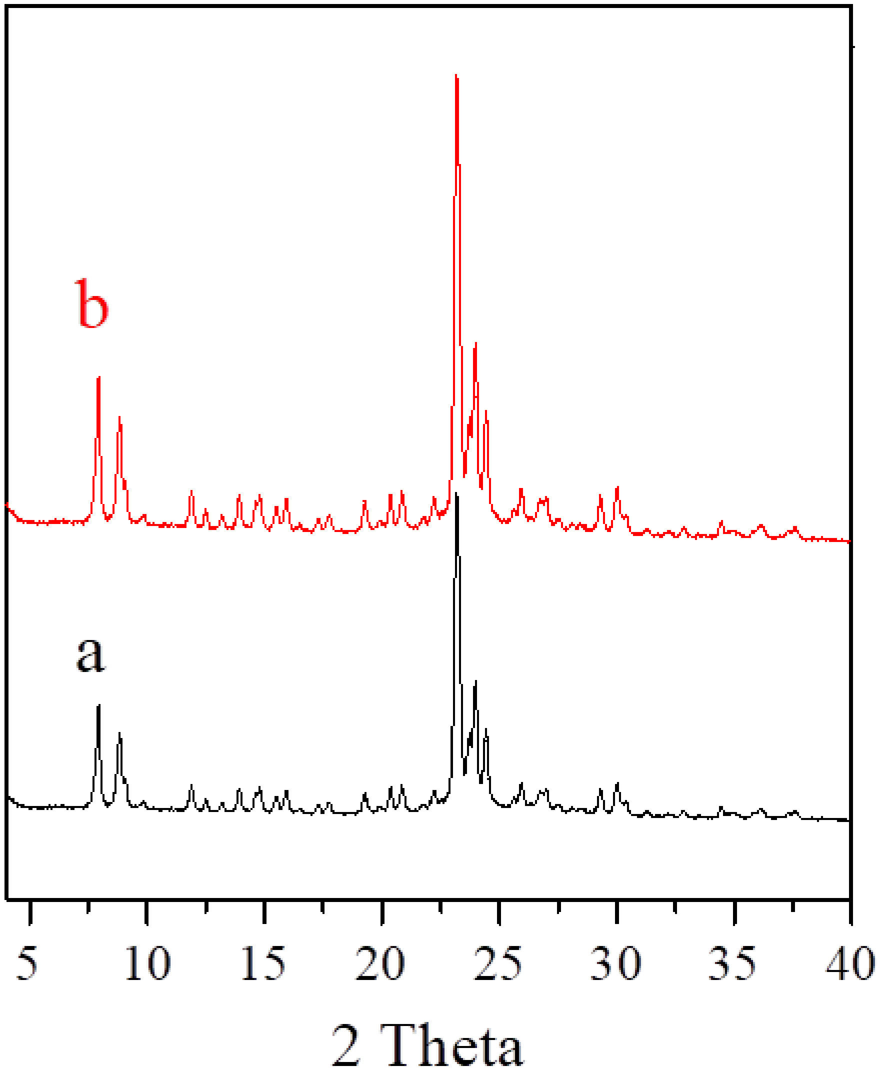

Figure S3. XRD patterns of the (a) C-S-1 (the Silicalite-1 seed) and (b) commercial Silicalite-1 samples.

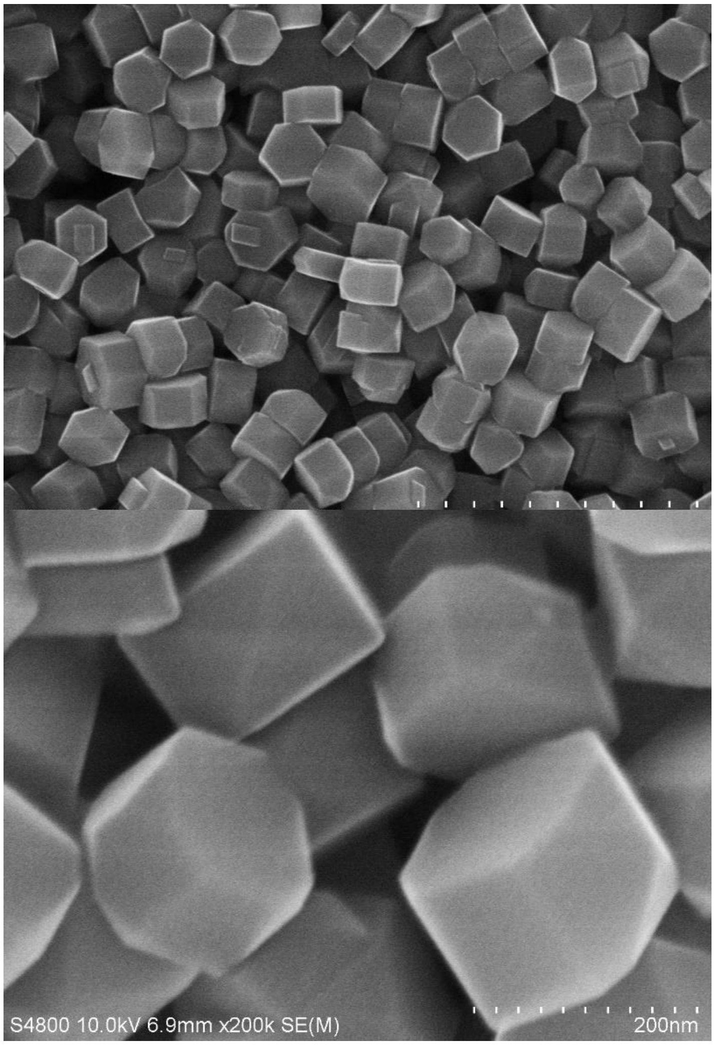

Figure S4. SEM images of C-S-1 (the Silicalite-1 seed) samples.

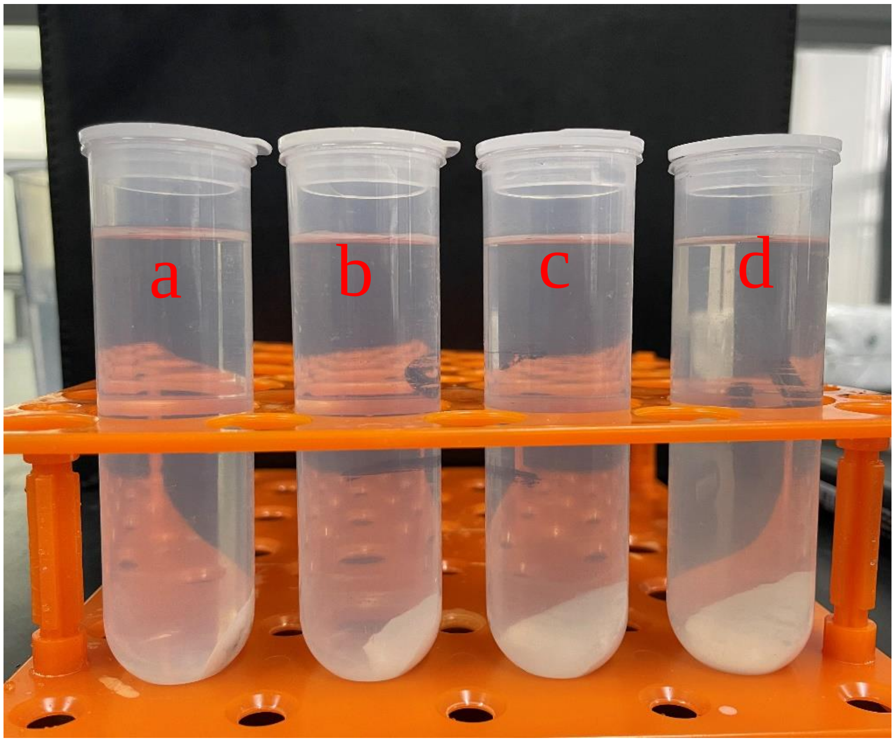

Figure S5. The photos of obtained samples after centrifugation with crystallization time at (a) 2, (b) 6, (c) 8, and (d) 10 min.

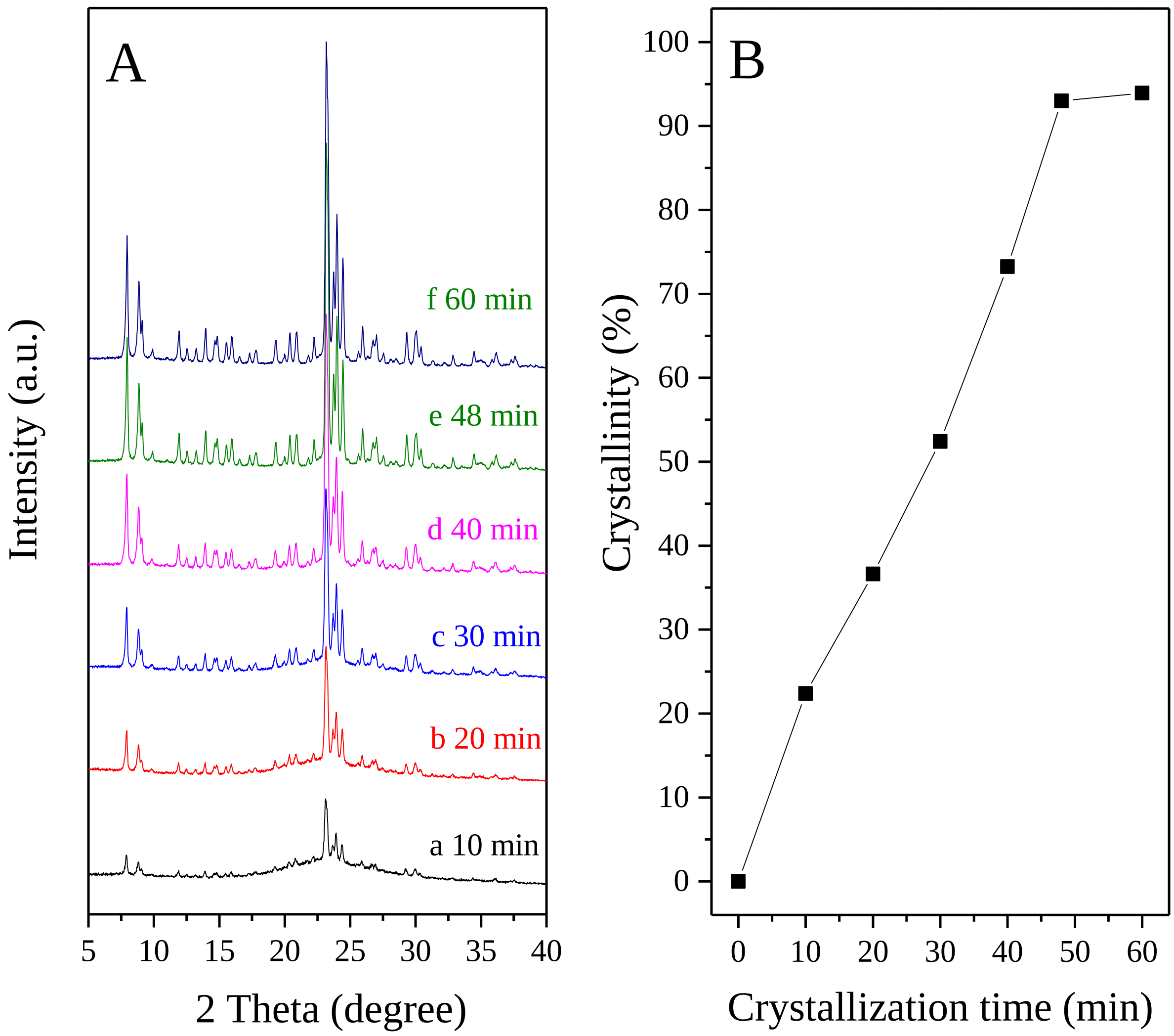

Figure S6 (A) XRD patterns of the samples with crystallization time at (a) 10, (b) 20, (c) 30, (d)48, and (e) 60 min; (B) Dependence of product crystallinity over crystallization time. (All the samples are synthesized with the H_2O/SiO_2 ratio at 15 and commercial Silicalite-1 zeolites are used as crystallinity baseline.)

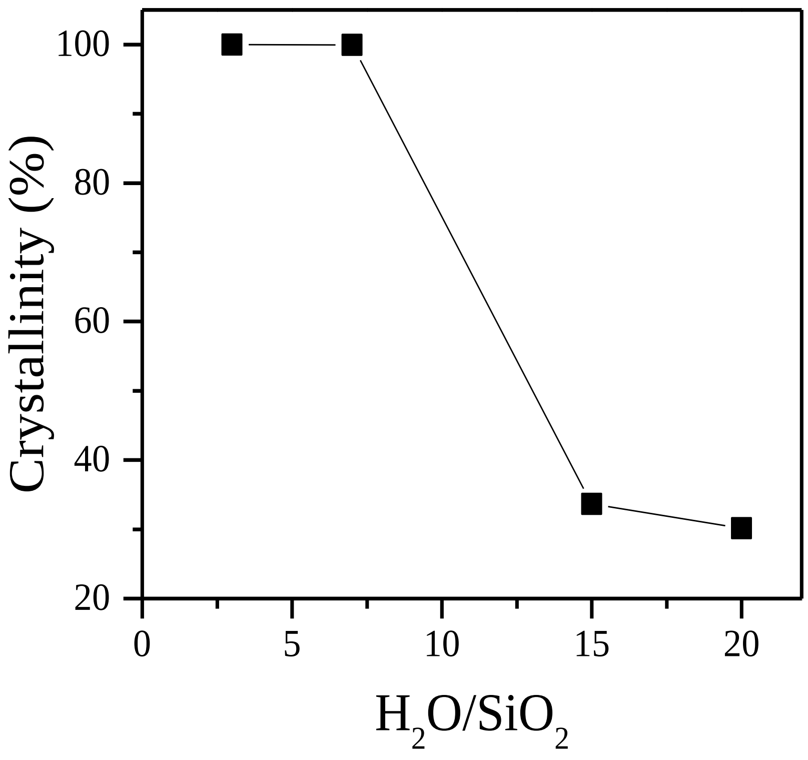

Figure S7. The crystallinity of zeolite samples with different H_2O/SiO_2 (all the samples are crystallized for 10min).

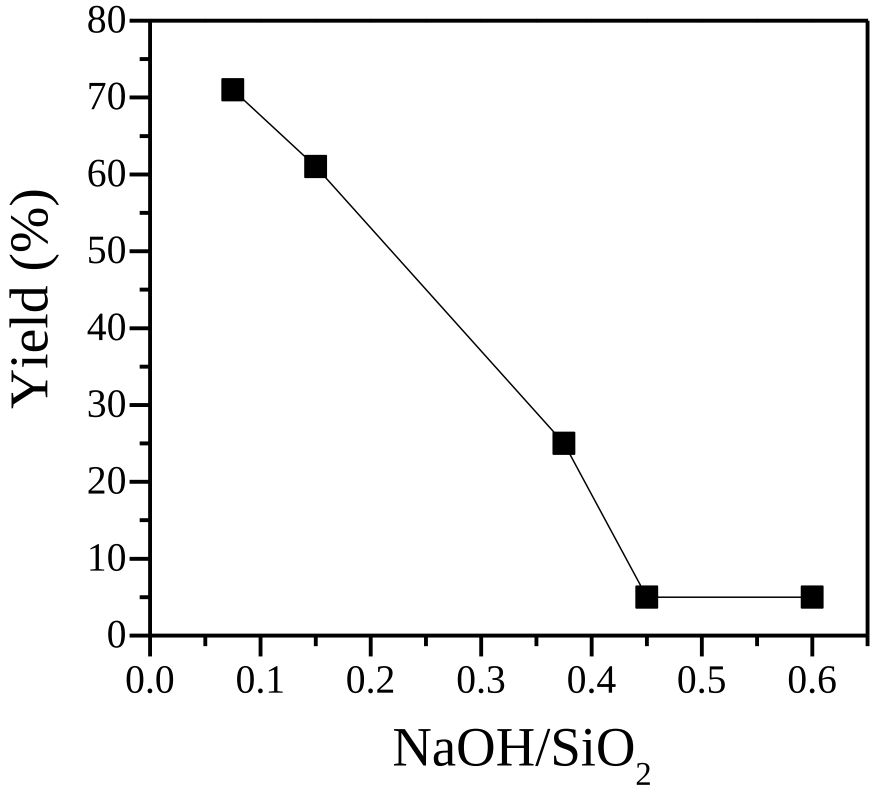

Figure S8. Zeolite product yields with different $NaOH/SiO_2$ (all these samples were crystallized for 10 min).

From this curve we could see the extra additional alkaline (NaOH) has great influence over the crystallization process and product yields of S-1-F. In this research, we could get the balance of short crystallization time and high zeolite yields with the $NaOH/SiO_2$ at 0.075 (extra addition of NaOH at 0.05g/1g SiO_2).
